# Assessment of Health-Related Quality of Life between People with Parkinson’s Disease and Non-Parkinson’s: Using Data Drawn from the ‘100 for Parkinson’s’ Smartphone-Based Prospective Study

**DOI:** 10.3390/ijerph15112538

**Published:** 2018-11-13

**Authors:** Xiaojing Fan, Duolao Wang, Bruce Hellman, Mathieu F. Janssen, Gerben Bakker, Rupert Coghlan, Amelia Hursey, Helen Matthews, Ian Whetstone

**Affiliations:** 1 Department of Epidemiology and Health Statistics, School of Public Health, Xi’an Jiaotong University Health Science Centre, Xi’an 710061, China; emirada@163.com; 2 Department of Clinical Sciences, Liverpool School of Tropical Medicine, Liverpool L3 5QA, UK; 3 uMotif Limited, London SE1 1JA, UK; Rupert@umotif.com; 4 Section Medical Psychology and Psychotherapy, Department of Psychiatry, Erasmus MC, 3015 GD Rotterdam, The Netherlands; mf.bas.janssen@googlemail.com; 5 EuroQol Research Foundation, 3068 AV Rotterdam, The Netherlands; bakker@euroqol.org; 6 Parkinson’s UK Research Directorate, London SW1V 1EJ, UK; ahursey@parkinsons.org.uk; 7 The Cure Parkinson’s Trust, London W1U 6TU, UK; helen@cureparkinsons.org.uk; 8 100 for Parkinson’s Data Access Committee, London SE1 1JA, UK; whetstone.home@gmail.com

**Keywords:** Parkinson’s disease, EQ-5D-5L, EQ visual analogue scale, smartphone, prospective study

## Abstract

*Background*: This study aims to assess the specific difference of the health-related quality of life between people with Parkinson’s and non-Parkinson’s. *Methods*: A total of 1710 people were drawn from a prospective study with a smartphone-based survey named ‘100 for Parkinson’s’ to assess health-related quality of life. The EQ-5D-5L descriptive system and the EQ visual analogue scale were used to measure health-related quality of life and a linear mixed model was used to analyze the difference. *Results*: The mean difference of EQ-5D-5L index values between people with Parkinson’s and non-Parkinson’s was 0.15 (95%CI: 0.12, 0.18) at baseline; it changed to 0.17 (95%CI: 0.14, 0.20) at the end of study. The mean difference of EQ visual analogue scale scores between them increased from 10.18 (95%CI: 7.40, 12.96) to 12.19 (95%CI: 9.41, 14.97) from baseline to the end of study. *Conclusion*: Data can be captured from the participants’ own smart devices and support the notion that health-related quality of life for people with Parkinson’s is lower than non-Parkinson’s. This analysis provides useful evidence for the EQ-5D instrument and is helpful for public health specialists and epidemiologists to assess the health needs of people with Parkinson’s and indirectly improve their health status.

## 1. Introduction

Parkinson’s disease (PD) is a degenerative movement disorder affecting 1–2% of the population aged over 60 years and is the second most common neurodegenerative disorder after Alzheimer’s disease [1,2]. It is a universal disorder, with an estimated global incidence of 4.5–19 per 100,000 population per year and it is expected that the number of people with PD will double by 2030 [3]. In the United Kingdom (UK), the annual incidence of PD is 12 per 100,000 population [4]; in Sweden, it is 19.7 per 100,000 [5] and in Norway it is 12.6 per 100,000 [6]. The core motor features of PD comprise combinations of bradykinesia, resting tremor, rigidity, flexed posture, ‘freezing’ and loss of postural reflexes [7]. It is a disabling condition with significant impact on health-related quality of life (HRQoL) [8,9], as well as the non-motor symptoms of PD such as dementia, sleep disturbances, depression and falls [10]. HRQoL is a multidimensional and comprehensive construct that assesses the perceived impact of health or disease on the physical, mental and social functioning [11]. Generally, the EQ-5D is the most popular generic measure of health status for clinical and economic appraisal [12,13].

Traditionally, research data is collected by health care professionals in hospitals, clinics and the community. However, novel data sources have now emerged with the widespread use of mobile phones [14]. Software applications are increasingly being used to consent participants, determine the eligibility of participants, run study tasks and provide reliable data without the need for participants to travel to study sites. Examples are as follows: a UK smartphone study examines the association between weather and pain [15]; an asthma symptom-tracking app uses GPS tracking to provide air quality updates in users’ locations [16]; an app collects data on gait, voice changes, balance, dexterity, and memory in subjects with Parkinson’s disease [17]; an app uses participants’ physical activity and lifestyle information as well as surveys to evaluate risks of cardiovascular disease [18]. In addition, Mulhern et al. (2014) showed that completing the EQ-5D using mobile phones produced equivalent results to more traditional methods such as paper-and-pencil, but with added benefits such as lessening the burden of data entry [19]; Bot et al. (2016) emphasized that data collection via mobile phones have the advantage of large enrollment and repeated measurements on many individuals and ultimately may lead to quantification of the ebbs-and-flows of Parkinson symptoms [20].

However, to our best knowledge, there is no empirical evidence based on smartphone data capture to assess the health-related quality of life for people with Parkinson’s. It is well-known that people with Parkinson’s disease’s health-related quality of life is lower than the non-Parkinson’s, but no specific data have been reported on the magnitude of the difference. Public health specialists, epidemiologists, and policy makers need the data to assess the health needs of society and indirectly improve health status.

This paper aims to fill this gap by using the EQ-5D measure. There are two key strengths in general. Firstly, the data were drawn from one of the largest smartphone-powered studies which enabled symptom tracking for people with Parkinson’s. Secondly, it is the first study in UK to use the EQ-5D-5L descriptive system and the EQ visual analogue scale (EQ VAS) to show the specific difference of health-related quality of life for people with Parkinson’s and non-Parkinson’s.

## 2. Materials and Methods

### 2.1. Data Source

**Figure 1:**
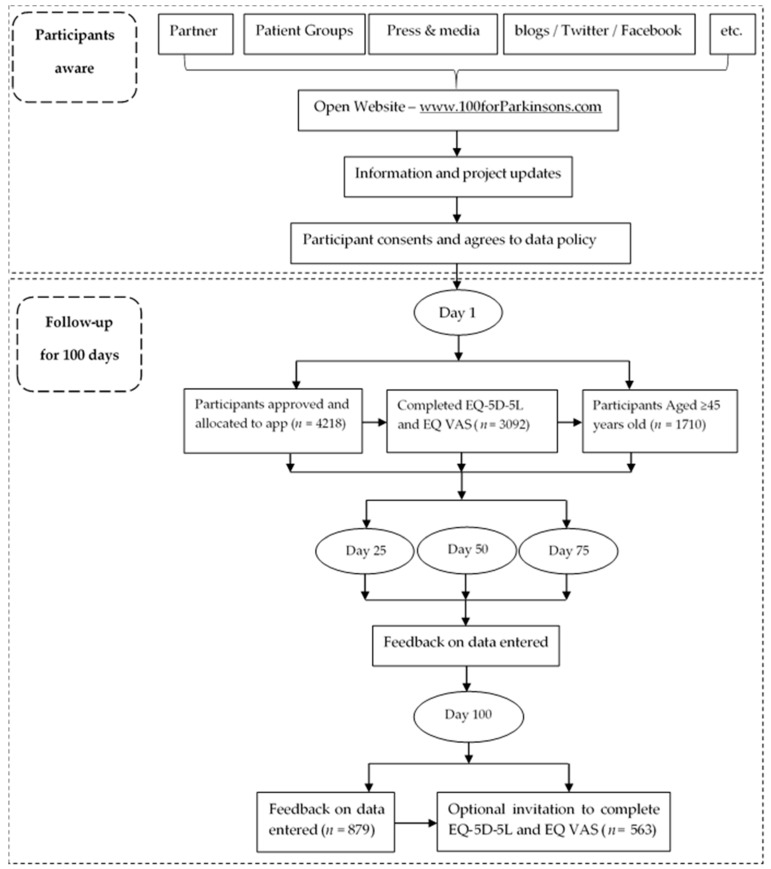
Participants flow chart of 100 for Parkinson’s project.

The data used for analysis constitutes one part of a dataset derived from the project named ‘100 for Parkinson’s’, an initiative of uMotif in conjunction with the Cure Parkinson’s Trust, Parkinson’s UK and the European Parkinson’s Disease Association. It was a smartphone-based study and followed the Strengthening the Reporting of Observational studies in Epidemiology (STROBE) guidelines for reporting observational studies [21]. This project was a ‘public facing’ project, focused on engaging patients to capture data, with all participants being invited to use the uMotif data capture app on their own Android or iOS mobile phones and/or tablets for 100 consecutive days from the day they register to use the app. They then received summary feedback based on the data they entered at 25, 50, 75 and 100 days. Participants used the uMotif app to record a range of data including: daily symptoms, questionnaires (EQ-5D, PDQ-8, NMS30) diaries, medications, cognitive and motor testing games, biometric results and—for those with devices—the opportunity to connect to wearable devices (i.e., Fitbit, Pedometers, Apple watch). Participants became aware of the project via the general press and media, blogs, Twitter, Facebook, etc. People with Parkinson’s were the primary participant group; the group of non-Parkinson’s were mainly interested people from the general public visitors and were recruited from the open website (http://www.100forparkinsons.com). To be eligible, participants were adults aged over 18, with the ability to read, write, and speak in English at levels of proficiency necessary to function daily at their jobs and/or daily living, and had access to a smartphone and/or tablet on a daily basis. There were no exclusion criteria applied. A total of 4218 participants consented to take part in this project, patients from 62 countries expressed an interest in taking part. Among these respondents, this paper is UK only and based on data from participants who completed the EQ-5D questionnaire and aged more than 45 years old (*n* = 1710). At baseline, 1050 People with Parkinson’s and 660 non-Parkinson’s finished the EQ-5D instruments; at end of study (day 100), 301 people with Parkinson’s and 262 non-Parkinson’s finished the EQ-5D instruments (Figure 1).

### 2.2. Research Ethics

The ‘100 for Parkinson’s’ study was conducted in accordance with the Declaration of Helsinki and received ethical approval from the Liverpool School of Tropical Medicine (No.15-050) and an ethics exemption from the New England IRB for participants from the USA (No.16-042). Consent was obtained by adapting E-Consent, a Participant Centered Consent (PCC) Toolkit developed by Sage Bionetworks. The process is similar to the standard consent process with the only difference being that a wet signature is not collected. All 100 for Parkinson’s participants provided e-consent for their de-identified data to be used in any future research projects that have been approved by the ‘100 for Parkinson’s Data Access Committee’. This current study received approval from the Data Access Committee for the analysis reported in this paper.

### 2.3. EQ-5D

EQ-5D is a standardized measure of health status developed by the EuroQol Group and includes the EQ-5D-5L descriptive system and the EQ visual analogue scale (EQ VAS) [22]. It is designed for self-completion, taking only a few minutes to complete. Firstly, the EQ-5D-5L asks patients to classify their health based on self-assessed levels of problems (no problems, slight problems, moderate problems, severe problems, and extreme problems or unable to perform) on five dimensions: mobility, self-care, usual activities, pain/discomfort and anxiety/depression. It contains 3125 possible health states defined by combining one level from each dimension, ranging from 11111 (full health) to 55555 (worst health). The numerals 1–5 have no arithmetic properties and should not be used as a cardinal score. A preference-based set of value set is used to calculate EQ-5D-5L index value from each health state because different country has a different value set according to the guidelines introduced by EuroQol Group. It is measured on a ‘0’ to ‘1’ scale where ‘0’ is defined as a health state equivalent to being dead and ‘1’ is full health. The EQ-5D-5L crosswalk value set for the United Kingdom was used, with index value ranges from −0.594 to 1 where ‘1’ is full health and −0.594 represents worst health [23]. Secondly, the EQ VAS asks respondents to rate their health on a visual analogue scale ranging from 0 (the worst imaginable health state) to 100 (the best imaginable health state). It is a quantitative measure to judge the health states of individual respondents. As sponsor of the ‘100 for Parkinson’s’ study, uMotif obtained approval from the EuroQol Research Foundation to use the EQ-5D instruments in the study.

### 2.4. Study Variables

The demographic data were self-reported by participants as part of the ‘100 for Parkinson’s’ study onboarding questionnaire completed within the uMotif data capture application. Participants in this study were drawn from two self-identified groups: People with Parkinson’s and non-Parkinson’s. They were followed from “day 1” to “day 100”, the EQ-5D questionnaires via smartphone were collected at the start of study and at the end of study, so the visit variable was categorized into two groups: at baseline and at end of study. Social-demographic characteristics considered in this study as confounding factors included gender (female or male), education (up to 16 years old means people stayed in school up to 16 years old, up to 18 years old means people stayed in school up to 18 years old, university/college/equivalent means the highest level of schooling person has completed), smoking status (No, never; No, but used to; Yes, occasionally/ weekly or Yes, daily) and alcohol use (No, never; Monthly or less; 2–4 times per month; 2–3 times per week; 4 or more times per week). In addition, two dummy variables were collected indicating whether participants had long-term health conditions diagnosed by a physician (such as hypertension, diabetes, heart problems, respiratory disease or cancer), and whether they live alone. These variables were selected based on the previous studies but constrained by the variables collected in the survey [24,25].

### 2.5. Statistical Analyses

The linear mixed model (LMM) was used to analyze the outcome variables, which includes both fixed and random effects. In this study, LMM was employed to analyze the difference of the HRQoL between people with Parkinson’s and non-Parkinson’s before and after 100 days when controlling for other confounding factors. The characteristics of the variables used are shown in Table 1. We estimated two mixed models for EQ-5D-5L value and EQ VAS score separately: Model 1 has group of participants (people with Parkinson’s and non-Parkinson’s), visit (at baseline and at end of study), and interaction between group and visit as fixed effects and subject as random effect. Model 2 is a model adding gender, education, smoking status, alcoholic drinking status, health condition and life style as covariates into model 1. From the above mixed models, the group differences in EQ-5D-5L value and EQ VAS score together with their 95%CIs at baseline and the end of the study were derived [26].

All questionnaires were checked for missing data and outliers, and cleaned prior to data analysis. Mean (standard deviation) or frequency (proportions) were used to describe the characteristics of the study variables where appropriate. Differences in variables between people with Parkinson’s and Non-Parkinson’s were compared by chi-squared test. The statistical analyses were performed using SAS 9.4 (SAS Institute, Cary, NC, USA). A two-tailed *p* value < 0.05 was considered statistically significant.

## 3. Results

### 3.1. Characteristics of the Study Population

At baseline, a total of 1710 people aged more than 45 years old, including 1050 (61.40%) people with Parkinson’s and 660 (38.60%) non-Parkinson’s completed the EQ-5D instruments via their smartphone with a mean (standard deviation, SD) age of 62.62 (7.47) years old and 59.69 (7.17) years old for people with Parkinson’s and non-Parkinson’s, respectively. The basic characteristics were shown in Table 1. After 100 days, 301 (53.46%) people with Parkinson’s and 262 (46.54%) non-Parkinson’s completed the EQ-5D questionnaire again.

### 3.2. Main Results

**Figure 2:**
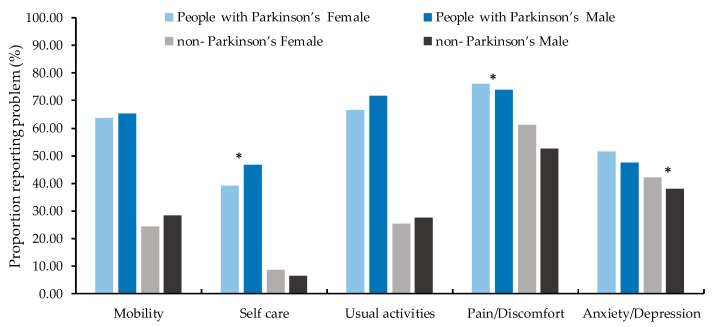
Frequency of reported problems for participants by gender at baseline (*n* = 1654). * means significant difference (*p* < 0.05) between two groups.

The health profile of participants at baseline is presented in Table 2 with the frequency of reported problems for each level by EQ-5D-5L dimension. People with Parkinson’s reported significantly (*p* ≤ 0.002) higher problems in mobility, self-care, usual activity, pain/discomfort and anxiety/depression than non-Parkinson’s. The most prevalent reported problems both for people with Parkinson’s and non-Parkinson’s were pain/discomfort: 74.86% and 58.48% respectively. Figure 2 showed for people with Parkinson’s, females had less frequency of reported problems on self-care (χ^2^ = 5.83, *p* = 0.016) but more frequency in pain/discomfort (χ^2^ = 4.33, *p* = 0.038) than males, there were no significant gender difference for mobility, usual activities and anxiety/depression (*p* > 0.05). For non-Parkinson’s, females reported more frequency of problem on anxiety/depression than males (χ^2^ = 8.54, *p* = 0.004) and there was no significant gender difference on other symptoms (*p* > 0.05).

**Figure 3:**
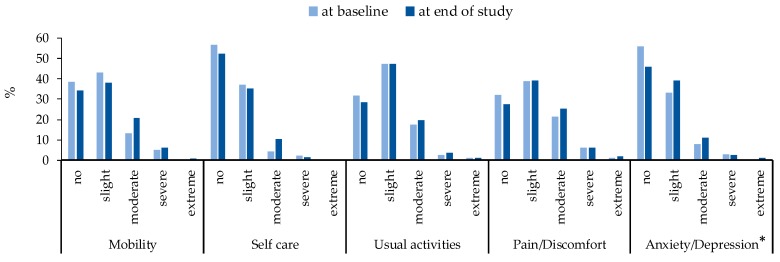
Changes of EQ-5D-5L from baseline to end of study for people with Parkinson’s (*n* = 301). * means significant difference (*p* < 0.05) among different level of anxiety/depression.

Table 3 summarized the EQ-5D-5L index values and EQ VAS scores at baseline and the end of study separately. Overall, the EQ-5D-5L values decreased significantly from 0.70 at baseline to 0.66 at the end of study for Parkinson’s (*p* = 0.042) and the EQ VAS scores decreased from 73.40 at baseline to 72.13 at the end of study for Parkinson’s without statistical support (*p* = 0.855), but they seem more stable for non-Parkinson’s (*p* > 0.05). According to Figure 3, for people with Parkinson’s, the percentage for no problems for anxiety/depression was decreased from baseline to the end of study while increased for slight and moderate problems (χ^2^ = 9.91, *p* = 0.042), there were no significant changes for mobility(χ^2^ = 8.91, *p* = 0.064), self-care (χ^2^ = 9.25, *p* = 0.055), usual activities(χ^2^ = 1.24, *p* = 0.871) and pain/discomfort(χ^2^ = 2.98, *p* = 0.562). Table 4 showed the mean differences of EQ-5D-5L values and EQ VAS scores between people with Parkinson’s and non-Parkinson’s at baseline and the end of study separately by four LMMs (before and after adjusting for subjects’ gender, education, smoking status, alcoholic drinking status, health condition and life style). After adjusting, the EQ-5D-5L value (confidence interval: CI) for people with Parkinson’s was 0.15 (95%CI: 0.12, 0.18) lower than non-Parkinson’s at baseline; the difference raised to 0.17 (95%CI: 0.14, 0.20) at the end of study. The mean difference of EQ VAS scores between people with Parkinson’s and non-Parkinson’s also increased from 10.18 (95%CI: 7.40, 12.96) to 12.19 (95%CI: 9.41, 14.97) from baseline to the end of study. This suggests the health-related quality of life for people with Parkinson’s was worse than non-Parkinson’s and that the difference between them increased with Parkinson’s disease progression.

## 4. Discussion

The EuroQol Group, which developed and owns the copyright on the EQ-5D, recommends that both the descriptive system of EQ-5D-5L and the EQ VAS should be used in the assessment of quality of life [27]. To the best of our knowledge, this is the first smartphone based prospective study reporting specific data on the difference of validity of EQ-5D-5L and EQ VAS between people with Parkinson’s and non-Parkinson’s in UK. Furthermore, 100 for Parkinson’s is the largest ever dataset gathered from Parkinson’s patients.

At the baseline, our study found the mean EQ-5D-5L values for people with Parkinson’s was 0.70, it was 0.83 for non-Parkinson’s. Because of the difference due to variation in categories of diseases, the population sampled or the value sets employed [28], we could not compare the mean EQ-5D-5L values for people in England with people in other places all over the world; however, we can find the difference of EQ VAS scores between them. In this study, The mean EQ VAS scores at the baseline was 73.40 for people with Parkinson’s, higher than stroke patients without coma (49.5) [29] and patients suffering from urinary incontinence (53.91) [30]; For non-Parkinson’s, the mean EQ VAS scores were 82.66, which were lower than the healthy people in Germany (91.5) [31] and Vietnam (87.4) [32] but higher than the EQ VAS scores in South Australian (78.6) [24], Spain (75.0), Italy (77.1), Korea (79.5) [33]. These conclusions show the health-related quality of life for people with Parkinson’s and non-Parkinson’s in the UK may not be low among patients with chronic disease and healthy populations in other countries.

Parkinson’s disease is the second most frequent neurodegenerative diseases and has progressive damage not only to motor symptoms but also to mental symptoms [34]. Our study finds the anxiety/depression for people with Parkinson’s became more serious after 100 days and quality of life for them became poorer. This is consistent with research on anxiety/depression may reduce quality of life in people with Parkinson’s disease because depression and anxiety may make patients have poorer response to receive treatment, more severe symptoms and higher functional impairment [35,36]. Therefore anxiety/depression should be considered in the future intervention strategies for people with Parkinson’s disease. In addition, our study estimated the specific difference of EQ-5D-5L values and EQ VAS scores between people with Parkinson’s and non-Parkinson’s after adjusting for confounders to make this conclusion generalizable. It is significant for clinicians and Parkinson’s carers or supporters to understand the condition of health-related quality of life for people with Parkinson’s with reference data. Public health specialists and epidemiologists may use the specific data to reflect the level of health-related quality of life for people with Parkinson’s and assess the health needs of them. In short, such data could be used by various stakeholders, to indirectly improve the health status of people with Parkinson’s.

For people with Parkinson’s, the prevalence of the most frequently reported EQ-5D-5L health state in our study was pain and discomfort, which was not surprising, considering that pain is a common symptom in Parkinson’s disease and can be found in two thirds of patients [37,38]. In addition, pain and discomfort was also the most frequently reported EQ-5D-5L health state for non-Parkinson’s participants which is similar to studies using traditional data collection surveys in England [39], South Australian [24], Germany [31] and Poland [40]. This equivalence indicates that a smartphone based prospective study may provide a viable alternative to traditional data collection methods. Furthermore, the ceiling effect was not high in this study with only 7.60% of people with Parkinson’s reporting a perfect health status, which is a lower ceiling effect than seen in a diabetic sample (nearly 1/3 of total sample) [41].

The current study has several limitations. Firstly, this is an observational study and the determinants of health-related quality of life included in this study are limited by the pre-specified questions in the surveys. There could be some potential unobserved confounding factors we did not control for in the linear mixed model. Secondly, EQ-5D questionnaires were administered in the fixed order, this may affect the proportion of missing answers. The desirable solution would be to present instruments in a random order. A third limitation is that the diagnosis of Parkinson’s was self-identified, without a formal diagnosis from a physician. A fourth limitation is the lack of control of the severity of Parkinson’s symptoms. A fifth limitation is our study is a prospective study and the follow-up bias inevitably influences the sample at the end of study. A final limitation is our study reports the changes of health-related quality of life for people with Parkinson’s based on quantitative study, more evidences based on the qualitative studies and randomized controlled trial are needed to clarify the functional change in the next research steps.

## 5. Conclusions

In summary, the smartphone based prospective study provides a viable data collection method to show that the health-related quality of life for people with Parkinson’s and non-Parkinson’s in the UK may not be low among patients with chronic disease and healthy population all over the world. The health-related quality of life for people with Parkinson’s is lower than non-Parkinson’s and the gap becomes more evident at the end of a prospective study. However, this is only a statistically significant change based on quantitative data; more evidence from qualitative studies are needed to clarify the functional change for people with Parkinson’s in the next research steps. This study provides useful literature on the EQ-5D, although the finding of health differences is to be expected. In addition, the data are helpful for understanding how low the health-related quality of life for people with Parkinson’s is compared to healthy population and are useful for public health specialists and epidemiologists to assess the health needs and indirectly improve their health status.

## Figures and Tables

**Table 1:** Characteristics of Parkinson’s and non-Parkinson’s participants at baseline (*n* = 1654).

Variables	Parkinson’s		Non-Parkinson’s		χ^2^	*p*
	*n*	%	*n*	%		
Gender					61.61	<0.001
Female	483	47.73	434	67.60		
Male	529	52.27	208	32.40		
Education					3.27	0.195
Up to 16 years old	166	16.40	87	13.55		
Up to 18 years old	109	10.77	64	9.97		
University/College/Equivalent	737	72.83	491	76.48		
Do you smoke tobacco?					1.36	0.714
No, never	638	63.04	392	61.06		
No, but used to	330	32.61	218	33.96		
Yes, occasionally/weekly	21	2.08	13	2.02		
Yes, daily	23	2.27	19	2.96		
How often do you have a drink containing alcohol?					1.36	0.714
No, never	155	15.32	65	10.12		
Monthly or less	185	18.28	101	15.73		
2–4 times per month	197	19.47	136	21.18		
2–3 times per week	248	24.50	184	28.66		
4 or more times per week	227	22.43	156	24.31		
Do you have other long-term health conditions?					3.35	0.067
No	578	57.11	335	52.18		
Yes	434	42.89	307	47.82		
Do you live alone?					0.68	0.411
No	876	86.56	547	85.20		
Yes	136	13.44	95	14.80		

**Table 2:** The distribution of reported EQ-5D-5L levels 1 to 5 by dimension at baseline (*n* = 1710).

Variables	Parkinson’s		Non-Parkinson’s		χ^2^	*p*
	*n*	%	*n*	%		
Mobility					244.51	<0.001
Level 1	374	35.62	489	74.09		
Level 2	424	40.38	120	18.18		
Level 3	190	18.10	32	4.85		
Level 4	56	5.33	18	2.73		
Level 5	6	0.57	1	0.15		
Self-care					245.69	<0.001
Level 1	597	56.86	607	91.97		
Level 2	347	33.05	35	5.31		
Level 3	87	8.29	9	1.36		
Level 4	17	1.62	8	1.21		
Level 5	2	0.18	1	0.15		
Usual Activity					305.39	<0.001
Level 1	323	30.75	487	73.79		
Level 2	465	44.29	124	18.79		
Level 3	202	19.24	32	4.85		
Level 4	47	4.48	12	1.82		
Level 5	13	1.24	5	0.7		
Pain/Discomfort					86.51	<0.001
Level 1	264	25.14	274	41.52		
Level 2	458	43.62	296	44.85		
Level 3	264	25.14	69	10.45		
Level 4	57	5.43	19	2.88		
Level 5	7	0.67	2	0.30		
Anxiety/Depression					17.37	0.002
Level 1	531	50.57	390	59.09		
Level 2	366	34.85	205	31.06		
Level 3	127	12.10	59	8.94		
Level 4	23	2.19	4	0.61		
Level 5	3	0.29	2	0.30		

level 1 response represents “no problems”, level 2 “slight problems”, level 3 “moderate problems”, level 4 “severe problems”, and level 5 “extreme problems” or “unable to perform.

**Table 3:** Summary of EQ-5D-5L values and EQ visual analogue scale (VAS) scores at baseline and the end of study (*n* = 563).

Summary	EQ-5D-5L		*p*	EQ-VAS		*p*
	At Baseline	At End of Study		At Baseline	At End of Study	
Parkinson’s			0.042			0.855
Mean	0.70	0.66		73.40	72.13	
SD	0.19	0.22		17.87	18.62	
Median	0.73	0.70		78.00	76.00	
Q1	0.62	0.58		62.00	65.00	
Q3	0.84	0.78		86.00	85.00	
Non-Parkinson’s			0.346			0.960
Mean	0.83	0.82		82.66	83.58	
SD	0.16	0.19		14.66	12.26	
Median	0.84	0.84		86.00	86.00	
Q1	0.75	0.74		79.00	77.00	
Q3	1.00	1.00		92.50	93.00	

SD: standard deviation; Q1 is equal to the 25th percentile of data; Q3 is equal to the 75th percentile of data.

**Table 4:** The mean differences of EQ-5D-5L values and EQ VAS scores between people with Parkinson’s and non-Parkinson’s: Results from linear mixed model analysis (*n* = 563).

EQ-5D	Model 1				Model 2			
	Mean Difference	95%CI		*p*	Mean Difference	95%CI		*p*
		Lower	Upper			Lower	Upper	
EQ-5D-5L values (Non-Parkinson’s vs. Parkinson’s)								
Baseline	0.13	0.10	0.17	<0.001	0.15	0.12	0.18	<0.001
End of study	0.16	0.12	0.18	<0.001	0.17	0.14	0.20	<0.001
EQ VAS scores (Non-Parkinson’s vs. Parkinson’s)								
Baseline	9.25	6.56	11.95	<0.001	10.18	7.40	12.96	<0.001
End of study	11.45	8.76	14.14	<0.001	12.19	9.41	14.97	<0.001

Linear mixed model was used to estimate the group differences at baseline and the end of study. In model 1, group of participants, visit, and group*visit are treated as fixed effects and subject as random effect. Model 2 is based on model 1 plus age of participants, gender, education, smoking status, alcoholic drinking status, health condition and life style. CI: Confidence Interval.
